# Systematic Review and Meta-Analysis of Cost-effectiveness of Rotavirus Vaccine in Low-Income and Lower-Middle-Income Countries

**DOI:** 10.1093/ofid/ofz117

**Published:** 2019-03-08

**Authors:** Sabbir Haider, Usa Chaikledkaew, Montarat Thavorncharoensap, Sitaporn Youngkong, Md Ashadul Islam, Ammarin Thakkinstian

**Affiliations:** 1 Mahidol University Health Technology Assessment (MUHTA) Graduate Program; 2 Social and Administrative Pharmacy Excellence Research Unit, Department of Pharmacy, Faculty of Pharmacy; 3 Section for Clinical Epidemiology and Biostatistics, Faculty of Medicine Ramathibodi Hospital, Mahidol University, Bangkok, Thailand; 4 Health Economics Unit, Ministry of Health and Family Welfare, Bangladesh, Bangladesh

**Keywords:** cost-effectiveness analysis, incremental net benefit, meta-analysis, rotavirus

## Abstract

**Background:**

Rotavirus causes morbidity and mortality in children particularly in low-income countries (LICs) and lower-middle-income countries (LMICs). This systematic review and meta-analysis aimed to assess cost-effectiveness of rotavirus vaccine in LICs and LMICs.

**Methods:**

Relevant studies were identified from PubMed and Scopus from their inception to January 2019. Studies were eligible if they assessed the cost-effectiveness of rotavirus vaccine in children in LICs and LMICs and reported incremental cost-effectiveness ratios. Risk of bias and quality assessment was assessed based on the Consolidated Health Economic Evaluation Reporting Standard checklist. Incremental net benefits (INBs) were estimated, and meta-analysis based on the DerSimonian and Laird method was applied to pool INBs across studies.

**Results:**

We identified 1614 studies, of which 28 studies (29 countries) were eligible and conducted using cost-utility analysis in LICs (n = 8) and LMICs (n = 21). The pooled INB was estimated at $62.17 (95% confidence interval, $7.12–$117.21) in LICs, with a highly significant heterogeneity (χ^2^ = 33.96; *df* = 6; *P* < .001; *I*^2^ = 82.3%), whereas the pooled INB in LMICs was $82.46 (95% confidence interval, $54.52–$110.41) with no heterogeneity (χ^2^ = 8.46; *df* = 11; *P* = .67; *I*^2^ = 0%).

**Conclusions:**

Rotavirus vaccine would be cost-effective to introduce in LICs and LMICs. These findings could aid decision makers and provide evidence for introduction of rotavirus vaccination.

Rotavirus is a viral pathogen that causes gastroenteritis with symptoms of fever, diarrhea, and emesis, which could lead to dehydration rapidly [1]. Rotavirus is among the leading causes of diarrhea among children aged <5 years, particularly in those aged <1 year. Globally, 111 million episodes of rotavirus gastroenteritis are estimated to occurr each year in children <5 years old [2]. According to the World Health Organization (WHO) estimate, about 215 000 (range, 197 000–233 000) children died of rotavirus infection globally in 2013 [3], and 85%–90% of these infections occurred in lower-middle-income countries (LMICs), particularly in Asia and Africa [4, 5].

Mortality and morbidity rates associated with rotavirus have decreased since implementation of 2 rotavirus vaccines since 2006 and 2009 [6]. This led to the WHO recommendation that rotavirus vaccines be included in national immunization programs where mortality rates in children were still high, especially in sub-Saharan Africa and South and Southeast Asia [7]. Two vaccines are available: the pentavalent (G1, G2, G3, G4, and P[8]) human-bovine reassortant vaccine (RV5; RotaTeq) and the monovalent (G1P) vaccine derived from an attenuated human strain (RV1; Rotarix) [4]. However, vaccine effectiveness varied according to the income of the countries, ranging from about 85% to 100% in high- or middle-income countries and from about 48% to 61% in low-income countries (LICs) [8].

A total of 21 LICs and 23 LMICs have implemented rotavirus vaccines in their national immunization programs [9]. However, those contemplating such implementation should consider not only the vaccine’s clinical effectiveness but also its cost-effectiveness, which is particularly important in LICs and LMICs. Nevertheless, economic evaluations of rotavirus vaccine are still limited in LICs and LMICs owing to lack of both local cost and clinical effectiveness data and the research capacity to conduct such evaluations [3, 10, 11]. Therefore, systematic reviews of published economic evaluations of rotavirus vaccine could be used as the evidence to guide decisions about vaccine policy.

Many individual studies have been conducted across the world to assess the cost-effectiveness of rotavirus vaccines, and these individual evidences have been summarized in 2 systematic reviews [3, 11]. However, the systematic review by Thiboonboon et al [11] mainly focused on methodological differences between economic studies conducted in high-income countries (HIC) and in LMICs, whereas the systematic review by Kotirum et al [3] provided only qualitative evidence without distinguishing LICs and LMICs. Neither review provided quantitative evidence of cost-effectiveness measured by the incremental cost-effectiveness ratio (ICER), the ratio of the cost difference between new and standard treatments to the clinical effectiveness difference between these treatments [12], which can be more useful for policy makers in LICs and LMICs.

Most results of economic evaluations are presented using ICERs. If the cost of new treatment is more expensive than the standard treatment but less effective, the new treatment is said to be dominated. Conversely, if the new treatment is less expensive but more clinically effective than the standard treatment, it is said to be dominant. However, if the new treatment is more expensive but also more clinically effective, the new treatment is said to be cost-effective if the ICER is less than the willingness to pay (WTP) for each individual country. Interpretation of the ICER is required for comparison with the WTP in the cost-effectiveness plane. The ICER itself is a ratio, its distribution may be not normal, and thus estimation of its confidence interval (CI) based on normal distribution may be invalid [13]. Therefore, an incremental net benefit (INB) has been developed, calculated by multiplying WTP times the difference in effectiveness subtracted from the difference in costs [14]. It is distributed normally based on the central limit theorem [12]. The new treatment is said to be cost-effective if the INB is positive [14, 15].

A meta-analysis for economic studies, called *comparative efficiency research,* has been developed to combine cost-effectiveness studies by pooling INB [12]. In light of the lack of economic evaluation study in resource-constrained countries (ie, LICs and LMICs), this method allows policy makers to make better decisions by pooling all available evidences (ie, INBs) from countries whose levels of income are epidemiologically similar. Nevertheless, it should be noted that health systems differ across countries, so transferability must be considered and evaluated before combining the cost-effectiveness results.

Therefore, the current systematic review and meta-analysis was conducted to assess whether the rotavirus vaccine was cost-effective by pooling INB data stratified by LICs and LMICs. Our results may provide useful information for policy decisions regarding rotavirus vaccine in LICs and LMICs. In addition, a lesson learned from our study should potential applications for further meta-analyses of cost-effectiveness.

## METHODS

### Data Sources and Searches

On 30 June 2017 we systematically searched Medline via PubMed and Scopus for relevant studies published globally since inception of the databases . We also conducted an updated search in 22 January 2019. The search terms and strategies were conducted for both databases based on the study’s targeted population, intervention, comparator, and outcomes, as described in detail in Supplementary A and B. The search results from both databases were merged, and duplicates were removed. The review was registered in PROSPERO, an international database of prospectively registered systematic reviews (registration no. CRD42017072587).

### Study Selection

Studies were determined to be eligible if they met the following criteria: (1) children <5 years of age as population of interest; (2) comparison of rotavirus vaccine with no vaccination; (3) outcome of interest: cost-effectiveness of rotavirus vaccination among the targeted population in the selected country; (4) study conducted in LICs or LMICs (Supplementary A).

We categorized countries according to the World Bank (WB) classifications, defining LICs and LMICs as countries with gross national income per capita of ≤$1005 and $1006–$3995, respectively [16]. There were 31 LICs and 52 LMICs according to the WB data accessed on 29 November 2017 [16]. WHO member states of are grouped in 6 regions: the African Region (AFR), Region of the Americas (AMR), South-East Asia Region (SEAR), European Region (EUR), Eastern Mediterranean Region (EMR), and Western Pacific Region (WPR) [17]. These countries are further divided into 14 epidemiological subregions—AFR-D, AFR-E, AMR-A, AMRO-B, AMR-D, EMR-B, EMR-D, EUR-A, EUR-B, EUR-C, SEAR-B, SEAR-D, WPR-A, and WPR-B [18, 19]. We also categorized countries according to the WHO epidemiological subregions, which are homogeneous in geographic locations, epidemiological status, and mortality stratum [19]. The 5 mortality strata—A, B, C, D, and E—was based on mortality rates for children <5 years old and the 15–59-year-old male population [18].

Two reviewers independently screened studies based on titles and abstracts. Full articles were retrieved if a decision could be not made based on the abstract. Studies that did not perform cost-utility analysis were excluded, and any disputes between the reviewers were solved by consensus between the 2.

### Data Extraction

We developed a standard data extraction form based on the Consolidated Health Economic Evaluation Reporting Standard (CHEERS) checklist [20]. Extracted information included country, study design, setting, characteristics of cost-effectiveness analysis (CEA) study, type of vaccine, and study outcomes. ICERs with its 95**%** CIs or/and results of ICER sensitivity analysis were extracted from individual studies. If the 95% CIs for ICERs were not reported, incremental cost and effectiveness data between rotavirus vaccination and no vaccination were extracted.

WTP data for each study setting were extracted from the study of the corresponding year, and the gross domestic product (GDP) per capita for the year 2016 was collected from the WB website [21]. We contacted authors of included studies to request additional data. However, these studies were excluded from meta-analysis if authors did not provide required data for pooling.

### Risk of Bias Assessment

We used the CHEERS checklist for assessing risk of bias [20]. We assessed based on these criteria: study perspective, description of comparator, time horizon, description of discounting of cost and outcome, description of model and with figures of model provided, clear reporting of study population, reporting ICER and its unit, sensitivity analysis, and disclosure of funding sources and any conflict of interest.

### Statistical Analysis

To standardize costing data, we adjusted all cost data to 2016 values using the consumer price index collected from the WB website [22]. We also collected country-specific GDP data for 2016 [21]. We calculated the INB for each study as ∆*E* × λ − ∆*C*, where ∆*E* is the difference in effectiveness, λ the threshold or GDP for each country in 2016, and ∆*C* as the difference in costs. For example, the INB for Fischer et al [23] was 195.48 = 0.1015 × 2170 − 24.99 (2016 consumer price index). The INB was then pooled across studies, using the fixed-effects model if there was no heterogeneity by an inverse variance method, as follows [12]:

$$\begin{array}{l} \begin{array}{ll} & INB_{P}=\frac{\sum_{i=1}^{S}w_{i}INB_{i}}{\sum_{i=1}^{S}w_{i}}\\ & w_{i}=\frac{1}{\mathrm{var}(INB_{i})}\\ & Var\left(INB\right)≅K^{2}\sigma_{\Delta E}^{2}+\sigma_{ICER}^{2} \end{array} \end{array}$$

None of the included studies reported variance of ∆*E*, so we therefore simulated ∆*E* data applying a Monte Carlo (MC) simulation. The number of simulation conducted corresponded to the number in the birth cohort for that country. For instance, in the study by Fischer et al [23], the birth cohort in Vietnam was found to be 1 639 000, and the data were simulated based on a ∆*E* of 0.1015 with 1 639 000 simulations. If the total number of particular birth cohort or population was not reported, then we simulated for 1000 times instead. After MC simulation, we calculated the variance of ∆*E*, which was 0.0071 for Fischer et al [23]. The variance of ICER was estimated from 95% CIs of ICER if reported, otherwise uncertainty analysis or sensitivity analysis was used as a proxy of 95% CIs, and variance was estimated accordingly. If heterogeneity were present, a random-effects model based on the DerSimonian and Laird method was applied, as follows:

$$\begin{array}{l} \begin{array}{ll} & INB_{P}=\frac{\sum_{i=1}^{S}w_{i}INB_{i}}{\left(\sum_{i=1}^{S}w_{i}+\tau^{2}\right)}\\ & \tau^{2}=\frac{Q-(S-1)}{\sum_{}w_{i}-\frac{\sum w_{i}^{2}}{\sum_{}w_{i}}} \end{array} \end{array}$$

The heterogeneity of INB between studies was assessed using the Cochran *Q* test and the *I*^2^ statistic, as follows:

$$\begin{array}{l} \begin{array}{ll} & Q={\sum_{i=1}^{S}w_{i}\left(INB_{i}-INB_{P}\right)}^{2}\\ & w_{i}=\frac{1}{\mathrm{var}(INB_{i})} \end{array} \end{array}$$

$$\begin{array}{l} I^{2}=\frac{(Q-S+1)\times 100}{Q} \end{array}$$

The degree of heterogeneity was considered low, moderate, and high if the *I*^2^ was < 25%, 25%–74%, or ≥75%, respectively, or if results of the *Q* test were significant (*P* < .10). Sources of heterogeneity were explored by fitting GDP, literacy rate, and vaccine coverage rate, one by one, into a meta-regression model. Each variable was considered a source of heterogeneity if regression coefficient was significant or if τ^2^ was decreased >50% after inclusion of that variable in the meta-regression model. A subgroup analysis was performed based on the epidemiological subregions of countries of the world. All analyses were performed using Stata software, version 14.0, and Microsoft Excel. Results were considered statistically significant for all analyses at *P* < .05 (2 sided).

## RESULTS

### Study Selection

**Figure 1.**
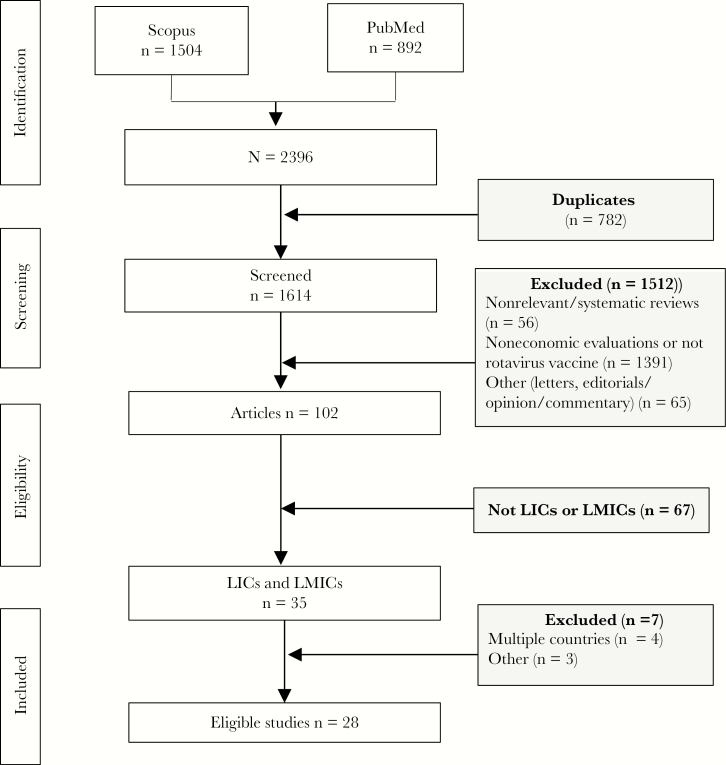
Flow diagram of selection of studies. Abbreviations: LICs, low-income countries; LMICs, lower-middle-income countries.

We identified 1504 records in Scopus and 892 records in PubMed; 782 were duplicates, leaving 1614 records for screening titles and abstracts (Figure 1). A total of 1512 articles were excluded, leaving 102 articles for further full-text reviews. Of these, 34 studies were from LICs or LMICs. Review of the full texts of these studies led to exclusion of 6 additional studies, finally resulting in 28 studies eligible for the systematic review.

### Characteristics of Studies

These 28 studies were published between 2005 and 2018. One study [24] used data from 2 independent countries; this was accounted for twice, resulting in a total of 29 included countries. The basic characteristics are described in Table 1. The 29 countries included 8 LICs and 21 LMICs, with 16 countries from Asia, 12 from Africa, and 1 from South America.

**Table 1.** General Characteristics of Included Studies

Authors (Year)	Country	WHO Region	Mortality Stratum	Target Population	Modelling Approach	Time Horizon, y	Discount Rate, Cost/Benefit, %	Perspective	Sensitivity Analysis	Most Sensitive Parameter
**Lower-Middle-Income Countries**										
Fischer et al 2005) [23]	Vietnam	WPR	B	Birth cohort	MS Excel based	5	NM/3	Govt, HCS	1 way, PSA	Price of vaccine
Isakbaeva et al (2007) [31]	Uzbekistan	EUR	B	Birth cohort	Static	5	NM/3	Societal	1 way	Mortality rate
Flem et al (2009) [32]	Kyrgyzstan	EUR	B	Birth cohort	MS Excel based	5	3/3	Societal, HCS	1 way	Price of vaccine
Ortega et al (2009) [33]	Egypt	EMR	D	Birth cohort	Decision tree	5	3/3	Govt, societal	1 way	Price of vaccine
Tate et al (2009) [34]	Kenya	AFR	E	Birth cohort	MS Excel based	5	NM/3	Societal	1 way	Vaccine efficacy
Wilopo et al (2009) [35]	Indonesia	SEAR	B	Birth cohort	Static	5	3/3	Societal, HCS	1 way	Vaccine efficacy against death
Kim et al (2009)[36]	Vietnam	WPR	B	Birth cohort	Markov	5	3/3	Societal, HCS	1 way, PSA	Price of Vaccine
Esposito et al (2011)[37]	India	SEAR	D	Birth cohort	MS Excel based	5	3/3	HCS	1 way	Vaccine efficacy
Jit et al (2011)[38]	Armenia	EUR	B	Birth cohort	Age structured	14	3/3	Govt, provider, societal	Scenario	NM
Smith et al (2011) [39]	Bolivia	AMR	D	Birth cohort	MS Excel based	5	3/3	Govt	1 way, PSA	Diarrhea-associated mortality rate
Abbott et al (2012) [40]	Ghana	AFR	D	Birth cohort	MS Excel based	5	3/3	Provider	1 way, multivariate	Price of vaccine
Tu et al (2012) [27]	Vietnam	EPR	B	Birth cohort	Consensus Rotavirus Vaccine	5	3/3	Provider, societal	1 way, scenario, PSA, other	Vaccine efficacy
Patel et al (2013) [41]	Pakistan	EMR	D	Birth cohort	Decision tree	5	3/3	Provider	1 way	Case-fatality ratio
Suwantika et al (2013) [25]	Indonesia	SEAR	B	Birth cohort	Consensus Rotavirus Vaccine	5	3/3	Provider, societal	1 way	Mortality rate
Suwantika et al (2013) [26]	Indonesia	SEAR	B	Birth cohort	Consensus Rotavirus Vaccine	5	3/3	Provider, societal	1 way	Breastfeeding pattern
Rheingans et al (2014) [42]	India	SEAR	D	Birth Cohort	MS Excel based	5	NM/3	NC	1 way, scenario, PSA, other	Vaccine administration cost
Sigei et al (2015) [24]	Kenya	AFR	E	Birth cohort	TRIVAC Excel based	20	3/3	Govt.	1 way, univariate, multivariate scenario	Incidence rate
Okafor et al (2017) [43]	Nigeria	AFR	D	Birth cohort	Markov	5	3/3	Govt, payer	1 way	Effectiveness of ORS and zinc
Pecenka et al (2017) [44]	Bangladesh	SEAR	D	Birth cohort	TRIVAC Excel based	10	3/3	Govt, societal	1 way, scenario	Delivery cost per dose
Rose et al (2017) [29]	India	SEAR	D		Dynamic	5	3/3	Societal, payer	1 way, multiway	Probability of receiving outpatient care given severe infection
Sarker et al (2018) [30]	Bangladesh	SEAR	D	Birth cohort	Decision tree	2	3/3	Societal, HCS	1 way, scenario	Price of vaccine
**Low-Income Countries**										
Berry et al (2010) [45]	Malawi	AFR	E	Birth cohort	Decision model -TreeAge Pro	2	3/3	HCS	1 way	Vaccine efficacy
Tate et al (2011) [23]	Uganda	AFR	E	Birth cohort	Decision tree	5	3/3	Govt, HCS	1 way	Program cost
Diop et al (2015) [47]	Senegal	AFR	D	Birth cohort	TRIVAC Excel based	20	3/3	Provider, societal	Scenario	Vaccine efficacy
Gargano et al (2015) [28]	Somalia	EMR	D	Birth cohort	Decision tree	1	NA	Provider	1 way	Vaccine administration cost (in routine immunization)
Ruhago et al (2015) [48]	Tanzania	AFR	E	Birth cohort	Markov	5	3/3	Provider	1 way	Vaccine effectiveness
Sigei et al (2015) [24]	Uganda	AFR	E	Birth cohort	TRIVAC Excel based	20	3/3	Govt, societal	1 way, other	Price of vaccine
Bar-Zeev et al (2016) [49]	Malawi	AFR	E	Birth cohort	TRIVAC Excel based	20	3/3	Govt, societal	Scenario	NM
Anwari et al (2017) [50]	Afghanistan	EMR	D	Birth cohort	UNIVAC Excel based	10	3/3	Govt, societal	Scenario	NM

Abbreviations: AFR, African Region; AMR, Region of the Americas; EMR, Eastern Mediterranean Region; EUR, European Region; Govt, Government; HCS, healthcare system; MS, Microsoft; NA, not applicable; NM, not mentioned; ORS, oral rehydration salt; PSA, probabilistic sensitivity analysis; SEAR, South-East Asia Region; WHO, World Health Organization.WPR, Western Pacific Region.

Mortality strata: Member states of WHO have been divided into 5 (A,B,C,D,E) mortality strata based on the levels of mortality in children under five years of age and in males 15–59 years old.

All studies performed cost-utility analysis; most studies used disability-adjusted life-years, and 3 studies [25–27] used quality-adjusted life-years. The time horizon of these ranged from 1 to 20 years, with a mode time horizon of 5 years. Most of the studies used a 3% discount rate for outcome, except 1 study [28] with only 1 year of time horizon (Table 1). A 2-dose rotavirus vaccine was used by 22 studies and a 3-dose vaccine by 7 studies. Among the investigators whose studies used 3-dose vaccine, Rose et al [29] mentioned the use of locally produced rotavirus vaccine and Sarker et al [30] used Rotavac vaccine, made in India. The vaccine efficacy, vaccine coverage, and price of vaccine varied from country to country, and rotavirus vaccine coverage was assumed to be the same as diphtheria, tetanus toxoids, and pertussis vaccine coverage in most countries (Table 2).

**Table 2.** Characteristics of Intervention and Economic Evaluation

Authors (Year)	Intervention	Comparator	No. of Doses	Vaccine Efficacy, %	Vaccine Coverage, %	Price per Dose, $	ICER (Base Case)	Unit of ICER	GDP per capita	Conclusion of EE study
**Lower-Middle Income Countries**										
Fischer et al (2005) [23]	Rotarix	NV	2	78–93	93–94	5	91	$/DALYs	550	Cost-effective
Isakbaeva et al (2007) [31]	RV	NV	2	93	98	1–12.5	489	$/DALYs	389	Cost-effective
Flem et al (2009) [32]	RV	NV	2	63–85	95	0.6	218	$/DALYs	490	Cost-effective and cost saving
Ortega et al (2009) [33]	Rotarix	NV	2	54.8–73.4	97	9.16	363	$/DALYs	1270	Very cost-effective
Tate et al (2009) [34]	RV	NV	2	85	71.5	0.5–10	27	$/DALYs	580	Very cost-effective
Wilopo et al (2009) [35]	RV	NV	2	84, 70, 76.5	80	7	120.46	$/DALYs	1560	Highly cost-effective
Kim et al (2009) [36]	Rotarix	NV	2	41 (21–62)	94	5	540	$/DALYs	580	Cost-effective
Esposito et al (2011) [37]	RV	NV	2	40–50	68	1.00	21.41	$/DALYs	1017	Very cost-effective
Jit et al (2011) [38]	Rotarix	NV	2	70.9	72.5	1–2	650	$/DALYs	3800	Very cost-effective
Smith et al (2011) [39]	RV	NV	2	85	90	9 (3–24)	143.84	$/DALYs	1758	Very cost-effective
Abbott et al (2012) [40]	Rotateq	NV	3	56.4–65	84.6	5	62.26	$/DALYs	695	Very cost-effective
Tu et al (2012) [27]	Rotateq	NV	3	63.9	93	5 (0.3 Gavi)	665	$/QALYs	1150	Cost-effective and cost saving
Patel et al (2013) [41]	RV	NV	2	48.3	85	5	149.5	$/DALYs	1182	Very cost-effective
Suwantika et al (2013) [25]	Rotateq	Breastfeeding, NV	3	70–84	94	5	149	$/QALYs	3495	Highly cost-effective
Suwantika et al (2013) [26]	Rotateq	NV	3	70–84	95	5	174	$/QALYs	3495	Highly cost-effective
Rheingans et al (2014) [42]	RV	NV	3	50	Various	1.25	139	$/DALYs	1490	Very cost-effective
Sigei et al (2015) [24]	RV1	NV	2	13.3–67	89	0.2 (2.5)	38	$/DALYs	942	Very cost-effective
Okafor et al (2017) [43]	RV1	IMCI, IMCI + RV, NV	2	69	70	10.3	514	$/DALYs	2178	Cost-effective
Pecenka et al (2017) [44]	RV	NV	2	45.2–48	92–94	2.19	82	$/DALYs	1190	Highly cost-effective
Rose et al (2017) [29]	RV (Indian made)	NV	3	30.4–53.6	72–88	1	56	$/DALYs	1445	Highly cost-effective
Sarker et al (2018) [30]	Rotavac	NV	3	40–85	40–96	1	740.27	$/DALYs	1466	Highly cost-effective
**Low-Income Countries**										
Berry et al (2010) [45]	Rotarix (mainly) and Rotateq	NV	2	19.2–68.3	87	5.5 (0.15 Gavi)	5.07	$/DALYs	312	Highly cost-effective
Tate et al (2011) [46]	RV	NV	2	20–70	83	0.15	3.96	$/DALYs	453	Highly cost-effective
Diop et al (2015) [47]	RV1	NV	2	30–74	94	0.5	92	$/DALYs	1032	Very cost-effective
Gargano et al (2015) [28]	Rotarix	NV	2	50	47	5.3	5.3	$/DALYs	112	Very cost-effective
Ruhago et al (2015) [48]	RV1	ORS + intravenous fluid, RV + ORS (IMCI), NV	2	57–85	93	8.4	43	$/DALYs	609	Low cost-effective compared with combinations of dairrhoea treatment
Sigei et al (2015) [24]	RV1	NV	2	33–67	84–89	0.2 (2.5)	34	$/DALYs	572	Very cost-effective
Bar-Zeev et al (2016) [49]	RV1	NV	2	40–64	88.55	2.5	19	$/DALYs	253	Highly cost-effective
Anwari et al (2017) [50]	Rotarix	NV	2	53.1	77.3	2.02	82	$/DALYs	562	Highly cost-effective

Abbreviations: DALYs, disability-adjusted life-years; EE, economic evaluation; GDP, gross domestic product; ICER, incremental cost-effectiveness ratio; IMCI, integrated management of childhood illness; NV, no vaccination; ORS, oral rehydration salt; QALYs, quality-adjusted life-years; RV, rotavirus.

The ICER (Base Case) means the ICER calculated by using mean values of all parameters inputted into the model according to model assumption.

### Sensitivity Analysis

All studies performed sensitivity analysis, 18 countries using single methods and the others using multiple methods of sensitivity analysis. One-way or univariate sensitivity analysis was used in 25 countries, scenario-based technique in 10, and probabilistic sensitivity analysis in 5. The price of vaccine was the most sensitive parameter in 7 countries, and vaccine efficacy or effectiveness was most sensitive in another 7. The other most sensitive parameters included vaccine administration or program cost, case-fatality ratio, and the incidence and effectiveness of oral rehydration therapy and zinc.

### Perspectives

Among the 29 countries, the societal perspective (n = 17) was used most often, followed by government (n = 11), provider (n = 10), healthcare system (n = 8), and payer (n = 2) perspectives.

### Cost-Effectiveness Results

Most studies (23 of 28) reported that the rotavirus vaccine was either very or highly cost-effective. Among 8 studies in LICs, 7 concluded that it was very or highly cost-effective and only 1 study concluded that it was less cost-effective than combination with diarrhoeal treatment. Among 21 studies in LMICs, 15 concluded that it was very or highly cost-effective, 6 that it was as cost-effective, and 2 also indicated that introducing rotavirus vaccine in the country would be cost saving (Table 2).

### Risk of Bias Assessment

**Figure 2.**
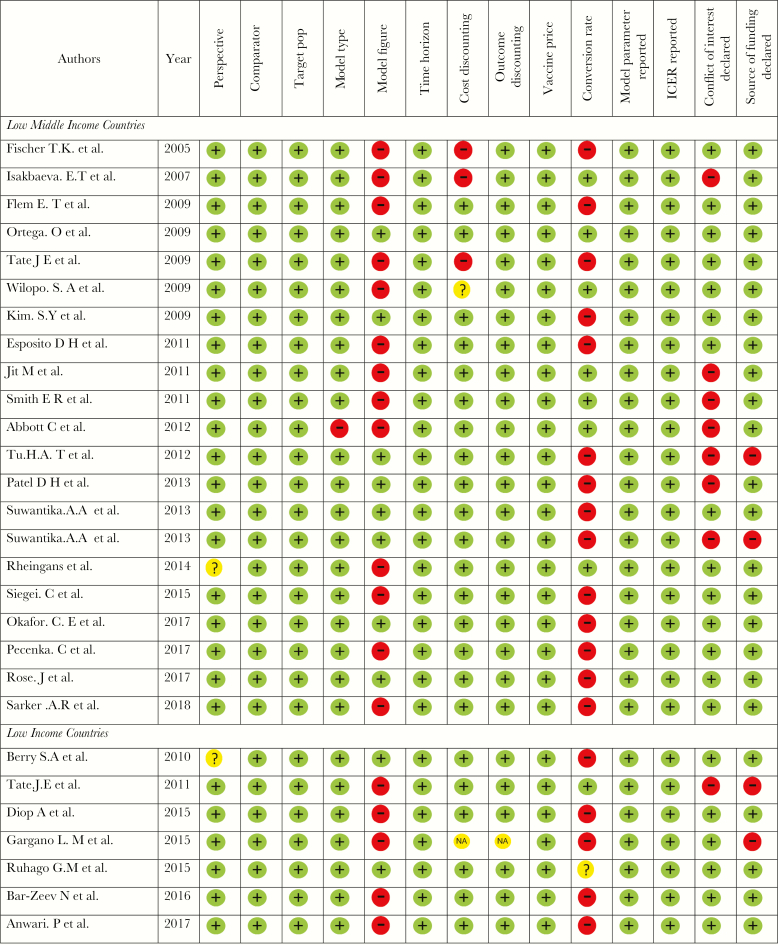
Risk-of-bias assessment among the studies reviewed. Plus signs represent yes (low risk of bias); minus signs, no (high risk of bias); question marks, results unclear (unclear risk of bias); and NA, not applicable.

The risk of bias or quality was assessed in all 28 studies, based on perspectives of the study, comparators of the intervention, target population of the intervention, type of analytical model used in the analysis, time horizon, discounting of both cost and outcome, date of price, parameter of model, and ICER. Results of assessments are presented in Figure 2.

### Pooling INBs by Level of Income

Among 29 countries, 5 studies from 6 countries did not reported either 95% CIs for ICER or the upper and lower limits of ICER from sensitivity analysis, 3 studies used quality-adjusted life-years for ICER, 1 study did not provide ∆*E*, leaving 19 countries eligible for pooling the INB data. These included 7 LICs and 12 LMICs.

**Figure 3.**
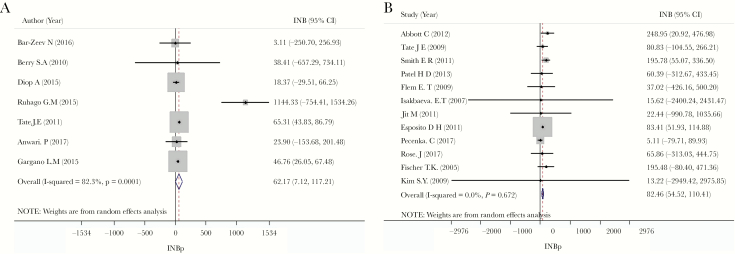
Pooled incremental net benefit (INB) of rotavirus vaccination by country’s income level. *A,* Low-income countries. *B,* Lower-middle-income countries. Abbreviation: CI, confidence interval.

Among the 7 LICs, the ICER ranged from $3.96 to $92 per disability-adjusted life-year, and the GDP per capita or the threshold ranged from $112 to $1032, with a median of $562. The INB was calculated for each country and then pooled across countries using a random-effects model based on the DerSimonian and Laird method, which yielded a pooled INB of 62.17 (95% CI, 7.12–117.2) with a degree of heterogeneity (*I*^2^) of 82.3% (χ^2^ = 33.96; *df* = 6; *P* < .01) (Figure 3A). This could be interpreted that in LICs, the INB of introducing rotavirus vaccination was equal to $62.17 per individual compared with no vaccination.

Among the 12 LMICs, the ICER ranged from $21.41 to $650 per disability-adjusted life-year. GDP per capita of country or the threshold varied from $389 to $3800. Individual INBs were plotted, and the pooled INB was 82.46 (95% CI, 54.52–110.41) with an *I*^2^ of 0% (χ^2^ = 8.46; *df* = 11; *P* = .67) (Figure 3B). In LMICs, the INB of introducing rotavirus vaccination, compared with no vaccination, was found to be $82.46 per individual.

**Figure 4.**
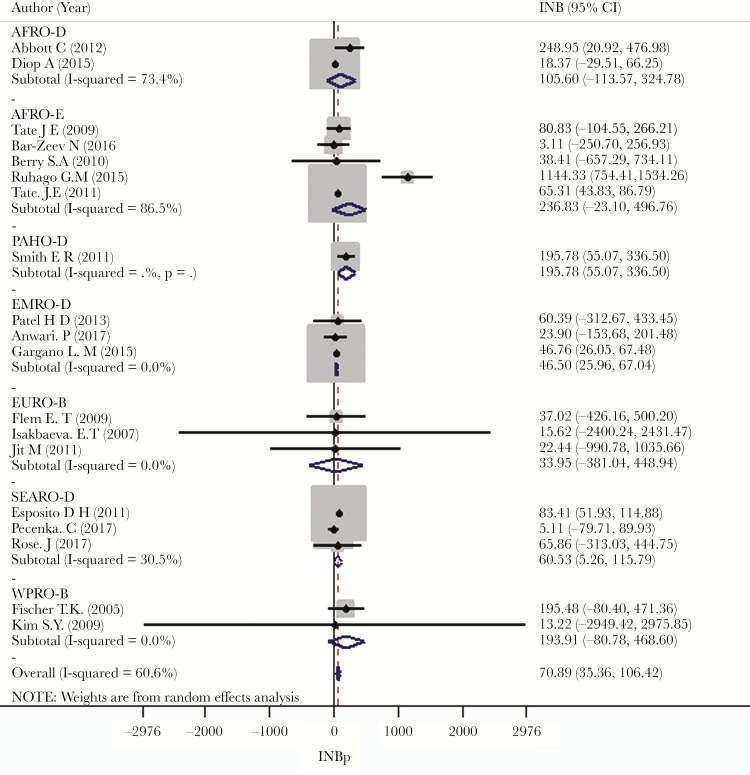
Pooling incremental net benefits (INBs) by World Health Organization epidemiological subregions. Abbreviations: AFR, African Region; AMR, Region of the Americas; CI, confidence interval; EMR, Eastern Mediterranean Region; EUR, European Region; South-East Asia Region; WPR, Western Pacific Region.

The studies reviewed were from the AFR (n = 11), AMR (n = 1), EMR (n = 3), EUR (n = 3), SEAR (n = 8), and WPR (n = 3) WHO regions. The countries were from 7 of 14 WHO epidemiological subregions. The INBs of these 7 subregions were heterogeneous, with *I*^2^ ranging from 0% to 86.5%. The pooled INBs were statistically significant in the EMR-D and SEAR-D subregions, at $46.5 (95% CI, $25.96–$67.04) and $60.53 ($5.26–$115.79), respectively (Figure 4).

## DISCUSSION

To the best of our knowledge, our study is the first to perform a systematic review and meta-analysis to assess whether the rotavirus vaccine was cost-effective, pooling INB data stratified by LICs and LMICs as well as WHO epidemiological subregions. A total of 28 studies from 29 countries, 8 LICs and 21 LMICs, were included. The pooled INBs were $62.17 (95% CI, $7.12–$117.21) in LICs and $82.46 ($54.52–$110.41) in LMICs. They were also significant in the EMR-D and SEAR-D regions, at $46.5 and $60.53, respectively.

A few systematic reviews on this topic had been conducted previously. In 2017, Kotirum et al [3] conducted a systematic review on global perspectives, including 104 studies; 21 studies were of LMICs, 9 of LICs, and 2 of both, but meta-analysis was not conducted [3]. Another systematic review, conducted by Thiboonboon et al [11] in 2016, focused on methodological comparison between resource-limited and resource-rich countries, including 14 studies from LMICs and 2 from LICs. However, these authors did not perform a meta-analysis [51]. The numbers of studies included in our review are compared with 2 previous reviews in Supplementary C.

Two studies published in 2017 conducted reanalysis of cost-effectiveness data by reconstructing ICER and making comparisons with different thresholds of WTP and different treatment costs, but they did not pool INBs [52, 53]. A study by May et al [54] did conduct meta-analysis of cost data from different studies with individual-level data.

Besides ICER, the INB, also known as net monetary benefit, is a valid tool for analyzing CEA. The INB is cost-effective if and only if it is compared with WTP (ie, λ × ∆*E* − ∆*C* > 0) [55]. The advantage of using INBs is there is a method of pooling that enables meta-analysis of CEA. Our meta-analysis found that the pooled INBs in both LICs and LMICs are >0, indicating that the introduction of rotavirus vaccine in LICs and LMICs may be cost-effective, and these LICs and LMICs will gain $62.17 and $82.46, respectively, by introducing rotavirus vaccine, compared with no vaccination. This indicates that the introduction of rotavirus vaccine may be more cost-effective in LMICs than in LICs. In addition, the pooled INB may also be cost-effective in EMR-D and SEAR-D.

It should be noted that different countries have different healthcare systems, service delivery systems are not homogeneous, and costs are measured from different perspectives. It is better if a country has specific CEA data, but not every country can conduct CEA owing to lack of expertise, data, and funding. Moreover, some countries have geographic, demographic, and epidemiological differences, and CEA results may even differ from region to region, making it difficult to conduct study within these countries. Despite these situations, there is a need for policy makers to use CEA results from other countries and apply them to their own setting. In reality, there is also a need for such transferability of cost-effectiveness data in 14 the epidemiological subregions in the world, based on similarities in geographic location, epidemiological status, and mortality stratum according to WHO. Policy makers can apply regional cost-effectiveness data in their local decision making [19].

To our knowledge, no meta-analysis has previously been conducted using INB data. In our study, we performed meta-analysis by LICs and LMICs and also subgroup analysis by WHO epidemiological subregions. Our result show that implementation of rotavirus vaccine is cost-effective in LICs and LMICs and in all epidemiological subregions. This evidence can be used to support introduction of the vaccine in national immunization program within these countries. Ours is a novel approach to the meta-analysis of economic evaluation studies. Results should be generalized only after taking into account local information (eg, perspective, time horizon, currency, and GDP/threshold) specific for each country. The method for pooling INB data should be explored more. It should be noted that the studies we analyzed were conducted from different perspectives and with different time horizons and variations in the results of sensitivity analyses. We used sensitivity analysis instead of 95% CIs for ICERs, and we also conducted MC simulation for ∆*E*. The variation in parameters both between and within countries also affects the CEA.

In conclusion, all studies included in this review conclude that introducing the rotavirus vaccine may be cost-effective, and the pooled INBs indicate that rotavirus vaccination may be cost-effective in both LICs and LMICs. Rotavirus vaccine is worth value for money in LICs and LMICs according to their WTP. Lessons learned from the current study may provide useful information to guide policy decisions on introducing rotavirus vaccine in LICs and LMICs, where economic evidence is limited, and suggest potential applications for further meta-analysis of cost-effectiveness. However, the cost-effectiveness results may be varied owing to differences in health systems, the values of parameters, and cost-effectiveness thresholds among these countries. Transferability must be considered and evaluated before synthesizing the cost-effectiveness results.

## Supplementary Data

Supplementary materials are available at *Open Forum Infectious Diseases* online. Consisting of data provided by the authors to benefit the reader, the posted materials are not copyedited and are the sole responsibility of the authors, so questions or comments should be addressed to the corresponding author.

ofz117_suppl_Supplementary_Material
