# Supplementary material for: Systematic Review and Meta-Analysis of Cost-effectiveness of Rotavirus Vaccine in Low-Income and Lower-Middle-Income Countries
Source: Open Forum Infect Dis. 2019 Mar 8;6(4):ofz117. doi: 10.1093/ofid/ofz117 (PMC6488528; doi:10.1093/ofid/ofz117)
Supplement: ofz117_suppl_Supplementary_Material [file ofz117_suppl_supplementary_material.docx]

**SUPPLEMENTARY**

**Supplementary A: PICO searching of the systematic review and meta-analysis**

|  | **Domain** | **Terms** |
| --- | --- | --- |
|  | Under 5 children | * |
|  | Rotavirus Vaccine | “Rotavirus vaccines” |
|  |  | “RotaTeq” |
|  |  | “RV5” |
|  |  | “Rotarix” |
|  |  | “RV1” |
|  |  | “Rotavirus” |
|  | No Vaccination | * |
|  | Cost-effectiveness | "economic evaluation" |
|  |  | "cost effectiveness " |
|  |  | “cost” |
|  |  | “Cost-effectiveness analysis” |
|  |  | "cost utility" |
|  |  | "cost benefit" |
|  |  | "Incremental Cost Effectiveness Ratio" |
|  |  | “ICER” |
|  |  | "Incremental Net Benefit" |
|  |  | “INB” |
|  |  | "Disability Adjusted Life Years" |
|  |  | “DALY” |
|  |  | "Quality Adjusted Life Years" |
|  |  | “QALY” |

*search term was not applied

**Supplementary B. Search Terms and Search Strategy of the systematic review and meta-analysis**

*Supplementary A1. PubMed Database*

Search ((((((((((((((((((cost AND OR ("cost effectiveness" OR ("cost effectiveness analysis" OR (CEA OR ("economic evaluation" OR ("cost utility" OR ("cost benefit" OR ("Incremental Cost Effectiveness Ratio") OR (ICER)) OR ("Incremental Net Benefit" OR (INB)) OR ("Disability Adjusted Life Years" OR (DALY OR ("Quality Adjusted Life Years" OR (QALY

AND ((((((((("rotavirus vaccines"[MeSH Terms] OR (RotaTeq OR (RV5 OR (Rotarix) OR (RV1) OR (rotavirus)

*Supplementary A2. Scopus Database*

( ( TITLE-ABS-KEY ( cost )   OR  ( TITLE-ABS-KEY ( "cost effectiveness" )   OR  ( TITLE-ABS-KEY ( "cost effectiveness analysis" )  OR  ( TITLE-ABS-KEY ( cea )

OR  ( TITLE-ABS-KEY ( "economic evaluation" )  OR  ( TITLE-ABS-KEY  ( "cost utility" )   OR  ( TITLE-ABS-KEY ( "cost benefit" )   OR  ( TITLE-ABS-KEY ( "Incremental Cost Effectiveness Ratio" )  OR  ( TITLE-ABS-KEY ( "ICER" )  OR  ( TITLE-ABS-KEY ( "Incremental Net Benefit" )  OR  ( TITLE-ABS-KEY ( "INB" )  OR  ( TITLE-ABS- KEY ( "DALY" )  OR  ( TITLE-ABS-KEY ( "Disability Adjusted Life Years" )  OR  ( TITLE-ABS-KEY ( "Quality Adjusted Life Years" )  OR  ( TITLE-ABS-KEY ( "QALY" ) )  AND

( ( TITLE-ABS-KEY ( "Rotavirus vaccines" )   OR  ( TITLE-ABS-KEY ( "RotaTeq" )   OR  ( TITLE-ABS-KEY ( "RV5" )  OR  ( TITLE-ABS-KEY ( "Rotarix" )  OR  ( TITLE-ABS-KEY ( "RV1" ) OR  ( TITLE-ABS-KEY ( "Rotavirus" ))

**Supplementary C. Comparison of economic studies including in this systematic review and previous reviews**

| **No.** | **Author Year** | **Journal** | **Our study** | **Kotirum et al.** | **Thiboonboon et al.** |
| --- | --- | --- | --- | --- | --- |
| ***Low Middle Income Countries*** | | | | | |
|  | Fischer et al. 2005 | J Infect Dis | √ | √ | √ |
|  | Isakbaeva et al. 2007 | Vaccine | √ | √ | √ |
|  | Flem et al. 2009 | J Infect Dis | √ | √ |  |
|  | Kim et al. 2009 | BMC Public Health | √ | √ | √ |
|  | Ortega et al. 2009 | J Infect Dis | √ | √ | √ |
|  | Rose et al. 2009 | BMJ |  | √ | √ |
|  | Tate et al. 2009 | J Infect Dis | √ | √ | √ |
|  | Wilopo et al. 2009 | Vaccine | √ | √ | √ |
|  | Esposito et al. 2011 | Clin Infect Dis | √ | √ | √ |
|  | Jit et al. 2011 | Vaccine | √ | √ | √ |
|  | Smith et al. 2011 | Vaccine | √ | √ | √ |
|  | Abbott et al. 2012 | Vaccine | √ | √ | √ |
|  | Connolly et al. 2012 | Pharmacoeconomics |  | √ |  |
|  | Tu et al. 2012 | Vaccine | √ | √ | √ |
|  | van Hoek et al. 2012 | PLoS One |  | √ | √ |
|  | Kotsopoulos et al. 2013 | Vaccine |  | √ |  |
|  | Patel et al. 2013 | Vaccine | √ | √ | √ |
|  | Suwantika et al. 2013 | BMC Public Health | √ | √ |  |
|  | Suwantika et al. 2013 | Vaccine | √ | √ |  |
|  | Megiddo et al. 2014 | Vaccine |  | √ |  |
|  | Rheingans et al.2014 | Vaccine | √ | √ |  |
|  | Okafor. C. E et al. 2017 | PLoS Negl Trop Dis | √ |  |  |
|  | Pecenka. C et al. 2017 | Vaccine | √ |  |  |
|  | Rose. J et al. 2017 | PLoS One | √ |  |  |
|  | Sarker .A.R et al. 2018 | Human Vaccines and Immunotherpeutics | √ |  |  |
| ***Low Middle Income Countries and Low Income Countries*** | | | | | |
|  | Verguet et al. 2013 | Vaccine |  | √ |  |
|  | Sigei et al. 2015 | Vaccine | √ | √ |  |
| ***Low Income Countries*** | | | | | |
|  | Berry et al. 2010 | J Infect Dis | √ | √ | √ |
|  | Tate et al. 2011 | Vaccine | √ | √ | √ |
|  | Diop et al. 2015 | Vaccine | √ | √ |  |
|  | Gargano et al. 2015 | Confl Health | √ | √ |  |
|  | Pecenka et al. 2015 | BMJ Open |  | √ |  |
|  | Ruhago et al. 2015 | Cost Eff Resour Alloc | √ | √ |  |
|  | Verguet et al. 2015 | Lancet Glob Health |  | √ |  |
|  | Bar-Zeev N et al. 2016 | Clinical Infectious Diseases | √ |  |  |
|  | Anwari. P et al. 2017 | Vaccine | √ |  |  |
